# Perceived Difficulty in Discharging Older Patients With Senility to Home or Care Facilities for End‐of‐Life Care: A Nationwide Cross‐Sectional Study of Hospital‐Based Geriatricians in Japan

**DOI:** 10.1002/jgf2.70181

**Published:** 2026-09-18

**Authors:** Teruhiko Imanaga

**Affiliations:** ^1^ Kanade Clinic Hasuda Saitama Japan

**Keywords:** discharge planning, end‐of‐life care, geriatrician, home medical care, long‐term care facility, senility

## Abstract

**Background:**

Senility has become an increasingly common cause of death in aging societies such as Japan. However, many patients still die in hospitals, suggesting challenges in transitioning end‐of‐life care to community settings. This study examined hospital‐based geriatricians' perceived difficulty in discharging patients with senility and associated physician‐related factors.

**Methods:**

I conducted a nationwide postal cross‐sectional survey of hospital‐based board‐certified geriatricians in Japan. Perceived difficulty in discharge and its reasons were assessed. A composite intervention frequency score was calculated based on four clinical practices used when patients were unable to maintain oral intake (hydration, central venous nutrition, nasogastric tube feeding, and percutaneous endoscopic gastrostomy). Ordinal logistic regression analyses were performed to examine the association between intervention frequency and perceived difficulty, adjusting for physician characteristics.

**Results:**

Of 409 respondents (response rate: 35.4%), 301 physicians with experience diagnosing senility were included. Most respondents reported experiencing difficulty in discharging patients with senility for end‐of‐life care to home (86.1% reported “always” or “often”) and to long‐term care facilities (72.5% reported “always” or “often”). Caregiver‐related factors were prominent barriers to home discharge, whereas facility‐related factors were more common for discharge to long‐term care facilities. Higher intervention frequency was associated with greater perceived difficulty in discharge to home (OR 1.13, 95% CI 1.01–1.26) and long‐term care facilities (OR 1.14, 95% CI 1.02–1.28).

**Conclusion:**

Hospital‐based geriatricians frequently perceive difficulty in discharging patients with senility. Barriers differ by setting, and physicians' intervention tendencies may influence discharge planning.

## 1 Introduction

Senility, defined as death resulting from age‐related frailty and progressive functional decline without a single dominant fatal disease, has become increasingly common in Japan's rapidly aging society. Deaths from senility have increased and now constitute the third leading cause of death [1]. The death certificate manual states: “use senility as the cause of death only in the case of so‐called natural death, which is an elderly person with no other obvious cause of death” [2]. Thus, senility is an officially recognized and increasingly used cause of death. Despite this growing recognition, approximately one‐third of older adults diagnosed with senility still die in hospitals [3], indicating persistent challenges in transitioning end‐of‐life care from acute care settings to the community.

Primary care plays a central role in end‐of‐life care; for example, a cohort study reported that 88% of patients accessed primary care services during the last 6 months of life, and primary care home visits were associated with reduced hospital use and enabling patients to remain at home [4]. A previous study concluded that initiatives are required to increase collaboration between primary care and hospital teams to improve continuity of care [5]. In this context, understanding the challenges faced by hospital physicians when arranging discharge for end‐of‐life care becomes crucial. If the degree and nature of difficulty perceived by hospital physicians are clarified, community‐based generalists may be better able to anticipate potential barriers, prepare appropriate care arrangements, and engage in earlier and more effective collaboration with hospital teams. Such shared understanding may contribute to smoother transitions of care and potentially reduce avoidable hospital deaths among older adults with senility.

However, little is known about how hospital physicians themselves perceive the difficulty of discharging patients with senility for end‐of‐life care, or which physician‐related factors are associated with such difficulties. Physicians' clinical approaches to end‐of‐life care may influence how they perceive discharge planning for patients with senility. In particular, a greater tendency to initiate medical interventions in situations such as loss of oral intake may reflect a more hospital‐centered, treatment‐oriented approach, which could be associated with increased perceived difficulty in arranging discharge for end‐of‐life care. However, empirical data examining these relationships remain limited. Board‐certified geriatricians are uniquely positioned to address these issues, as they are frequently involved in diagnosing senility, determining goals of care for frail older adults, and coordinating discharge planning in hospital settings. Clarifying their perspectives may therefore provide important insights into modifiable factors that influence end‐of‐life transitions from hospitals to the community.

The aims of this study were to examine the extent to which hospital‐based geriatricians perceive difficulty in discharging patients with senility for end‐of‐life care to home or long‐term care facilities, to describe the reasons for such perceived difficulties, and to explore physician‐related factors, particularly medical intervention tendencies.

## 2 Methods

### 2.1 Participants

I conducted a national survey targeting all 1196 geriatric specialists with the Japan Geriatrics Society as of January 2025, whose primary affiliation was a hospital. I carried out an anonymous survey using a questionnaire distributed via postal mail to 1196 participants between March 1 and March 31, 2025. The questionnaire included items on physician demographics, whether the physician had ever diagnosed senility in hospital settings, difficulty in discharging patients with senility for end‐of‐life care to home or long‐term care facilities, and the reasons for such perceived difficulties. For the present analysis, I included only physicians who had experience diagnosing patients with senility in hospital settings.

### 2.2 Questionnaire

These items included gender, years of experience as a physician, primary practice setting (general ward, long‐term care ward, rehabilitation ward, community‐based comprehensive care ward, or other), experience with home medical care (full‐time, part‐time, or none). To explore physicians' clinical decision‐making in the care of patients with senility, respondents were asked how frequently they performed specific medical interventions when patients with senility became unable to maintain oral intake. These interventions included intravenous or subcutaneous hydration, central venous nutrition, nasogastric tube feeding, and percutaneous endoscopic gastrostomy. Each item was rated on a four‐point Likert scale (1 = never to 4 = always).

Perceived difficulty in discharging patients with senility to home or long‐term care facilities for end‐of‐life care was assessed using a four‐point Likert scale (1 = never, 2 = rarely, 3 = often, 4 = always). For discharge to home, physicians were asked, “How often do you feel that it is difficult to discharge patients with senility who had been living at home before hospitalization to home for end‐of‐life care?” For discharge to long‐term care facilities, they were asked, “How often do you feel that it is difficult to discharge patients with senility who had been living in long‐term care facilities before hospitalization to long‐term care facilities for end‐of‐life care?” This single‐item measure was developed for this study and has not been formally validated as a psychometric instrument. The outcome items were designed to assess whether patients with senility could return to the place where they had lived before hospitalization and receive end‐of‐life care there.

Respondents who reported experiencing perceived difficulty in discharge (“always,” “often,” or “rarely”) were also asked to select applicable reasons for perceived difficulty from a list of predefined options (multiple responses allowed). Respondents who selected “never” or did not respond to the question on perceived difficulty were not asked to provide reasons. The response options were developed with reference to prior studies [6, 7]. The predefined response options are presented in Table S1.

The selection of variables related to home medical care experience and intervention frequency was guided by a priori clinical hypothesis that physicians' familiarity with home‐based end‐of‐life care and their tendency to initiate medical interventions might influence perceived discharge difficulty.

### 2.3 Ethical Considerations

The cover page of the questionnaire provided an outline of the survey, its purpose, privacy protection, and contact information. Informed consent was obtained via a checkbox on the cover page. This study was approved by the Ethical Review Committee of the Japanese Association for Home Care Medicine.

### 2.4 Statistical Analysis

Responses to the questions were summarized using standard descriptive statistics. To explore whether physicians' experience with home medical care was associated with differences in the reasons for perceived difficulty, I additionally examined the distribution of these reasons according to previous experience with home medical care. For each reason for perceived difficulty, differences between physicians with and without previous home medical care experience were assessed using Fisher's exact test. Statistical significance was determined using Bonferroni‐adjusted thresholds of 0.0056 for discharge to home and 0.0063 for discharge to long‐term care facilities. A composite intervention frequency score (range: 4–16) was calculated by summing the four intervention items and was entered into the regression model as a continuous variable, with higher scores indicating a greater tendency to initiate medical interventions. The internal consistency of the four intervention items was assessed using Cronbach's alpha, which was 0.70, indicating acceptable internal consistency [8].

To examine the association between intervention frequency and perceived difficulty in discharge, ordinal logistic regression analysis was performed. Perceived difficulty was treated as an ordinal outcome variable. Analyses were conducted separately for perceived difficulty in discharging patients to home and to long‐term care facilities. Cases with unanswered questions were excluded from the analyses, and complete‐case analysis was performed. First, univariable ordinal logistic regression analyses were conducted. Subsequently, multivariable ordinal logistic regression analysis was performed with adjustment for sex, years of clinical experience (entered as a continuous variable), primary practice setting (general ward vs. other), and experience with home medical care (full‐time or part‐time practice vs. none), which was entered as a binary variable. The proportional odds assumption was assessed using the test of parallel lines.

Sensitivity analyses were performed using two approaches. First, the “rarely” and “never” categories of perceived difficulty were collapsed into a single category, resulting in a three‐category outcome, and the ordinal logistic regression models were repeated using the same covariates as in the primary analysis. Second, missing data were handled using multiple imputation with 20 imputed datasets, and estimates were combined using Rubin's rules.

A two‐sided *p* value < 0.05 was considered statistically significant. All analyses were conducted using SPSS version 31 (IBM Corp., Armonk, NY, USA) and R version 4.5.1 (R Foundation for Statistical Computing, Vienna, Austria).

## 3 Result

Out of the 1196 geriatric specialists that originally dispatched the questionnaires, 39 geriatric specialists had to return the mail due to unknown addresses. The remaining 1157 questionnaires yielded 409 valid responses (response rate: 35.4%). Of the respondents, 301 physicians who had experience diagnosing patients with senility in hospital settings were included in the analysis.

The characteristics of the respondents are shown in Table 1. The mean years of physician experience was 28.0 (±11.6 SD), and the majority were male (79.4%). Most respondents primarily practiced in general wards (62.1%). Regarding experience with home medical care, 41.9% had no experience, while 25.2% and 32.6% had full‐time and part‐time experience, respectively. In terms of clinical practice, 86.7% of respondents reported “always” or “often” performing intravenous or subcutaneous hydration, whereas the corresponding proportions were 17.2% for central venous nutrition, 32.6% for nasogastric tube feeding, and 21.3% for percutaneous endoscopic gastrostomy.

**TABLE 1:** Background of the respondents (*n* = 301).

	*n*	%
Years of experience as a physician, mean (SD)	28.0 year (±11.6)	
Gender		
Male	239	79.4
Female	60	19.9
No response	2	0.7
Primary practice setting		
General ward	187	62.1
Long‐term care medical facility	31	10.3
Rehabilitation ward	16	5.3
Community‐based integrated care ward	22	7.3
Others	43	14.3
No response	2	0.7
Experience with home medical care		
None	126	41.9
Full‐time	76	25.2
Part‐time	98	32.6
No response	1	0.3
Medical interventions		
Intravenous or subcutaneous hydration		
Always	63	20.9
Often	198	65.8
Rarely	34	11.3
Never	5	1.7
No response	1	0.3
Central venous nutrition		
Always	1	0.3
Often	51	16.9
Rarely	151	50.2
Never	97	32.2
No response	1	0.3
Nasogastric tube feeding		
Always	3	1.0
Often	95	31.6
Rarely	148	49.2
Never	54	17.9
No response	1	0.3
Percutaneous endoscopic gastrostomy		
Always	2	0.7
Often	62	20.6
Rarely	154	51.2
Never	82	27.2
No response	1	0.3

**FIGURE 1:**
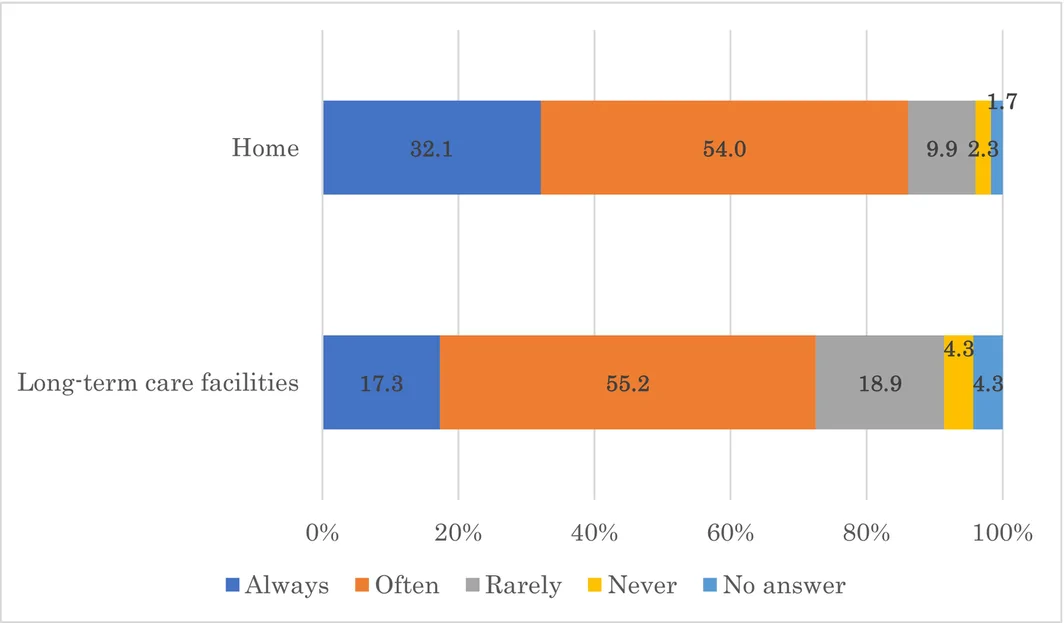
Perceived difficulty in discharging patients with senility for end‐of‐life care.

Responses regarding perceived difficulty in discharging patients with senility for end‐of‐life care were as follows. For discharge to home, 32.1% reported “always” experiencing difficulty and 54.0% reported “often,” whereas 9.9% reported “rarely” and 2.3% “never,” with 1.7% missing responses. For discharge to long‐term care facilities, 17.3% reported “always” and 55.2% “often,” while 18.9% reported “rarely” and 4.3% “never,” with 4.3% missing responses (Figure 1).

**FIGURE 2:**
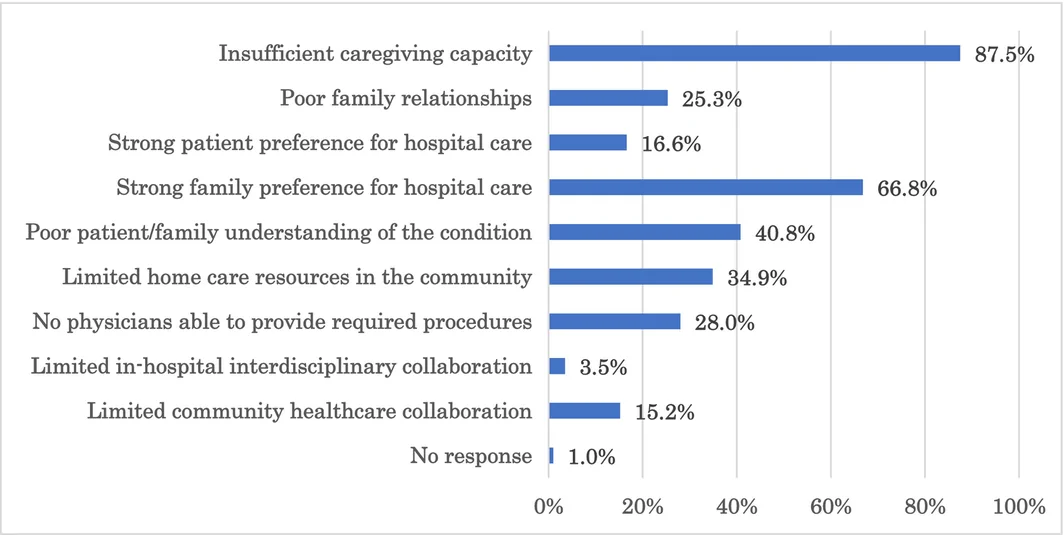
Reasons for difficulty in discharging patients with senility to home for end‐of‐life care (*n* = 290, multiple responses). Reasons for difficulty were assessed only among respondents reporting any perceived difficulty (“always,” “often,” or “rarely”); respondents selecting “never” or with missing responses were excluded.

**FIGURE 3:**
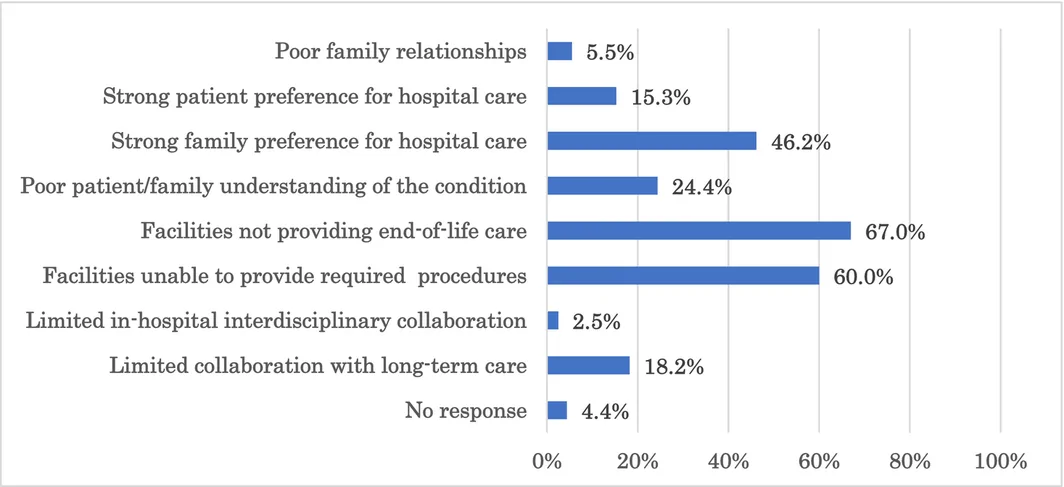
Reasons for difficulty in discharging patients with senility to long‐term care facilities for end‐of‐life care (*n* = 275, multiple responses). Reasons for difficulty were assessed only among respondents reporting any perceived difficulty (“always,” “often,” or “rarely”); respondents selecting “never” or with missing responses were excluded.

Figure 2 shows the reasons for difficulty in discharging patients with senility to home for end‐of‐life care. The most commonly reported reason was “insufficient caregiving capacity” (87.5%), followed by “strong family preference for hospital care” (66.8%) and “poor patient/family understanding of the condition” (40.8%). Figure 3 shows the reasons for difficulty in discharging patients with senility to long‐term care facilities. The most commonly reported reason was “facilities not providing end‐of‐life care” (67.0%), followed by “facilities unable to provide required procedures” (60.0%) and “strong family preference for hospital care” (46.2%). The reported reasons for perceived difficulty did not differ significantly according to physicians' experience with home medical care, either for discharge to home or to long‐term care facilities (Table S2).

To examine the association between intervention frequency and perceived difficulty in discharge, ordinal logistic regression analyses were performed. Complete‐case analysis was performed, including 290 of 301 physicians for discharge to home and 283 for discharge to long‐term care facilities. The results of the univariable ordinal logistic regression analyses are shown in Table S3. The composite intervention frequency score was significantly associated with greater perceived difficulty in both discharge to home (odds ratio [OR]: 1.13, 95% confidence interval [CI]: 1.01–1.26, *p* = 0.04) and discharge to long‐term care facilities (OR: 1.14, 95% CI: 1.02–1.28, *p* = 0.03), whereas physician sex, years of experience, the primary practice setting, and experience with home medical care were not significantly associated.

The results of the multivariable ordinal logistic regression analyses are shown in Table 2. After adjusting for physician sex, years of experience, the primary practice setting, and experience with home medical care, the composite intervention frequency score remained significantly associated with greater perceived difficulty in both discharge to home (OR 1.13, 95% CI 1.01–1.26, *p* = 0.04) and discharge to long‐term care facilities (OR 1.14, 95% CI 1.02–1.28, *p* = 0.03). The proportional odds assumption was assessed using the test of parallel lines and was not violated for either model (*p* = 1.00 for discharge to home and *p* = 0.42 for discharge to long‐term care facilities).

**TABLE 2:** Results of multivariable ordinal logistic regression analyses.

Variable	*p*	Odds ratio	95% confidence interval
Perceived difficulty in discharging patients to home (*n* = 290)			
Years of experience as a physician	0.45	0.99	0.97–1.01
Male	0.60	1.17	0.65–2.10
General ward as the primary practice setting	0.83	1.21	0.74–1.96
Experience with home medical care	0.30	0.78	0.49–1.24
Composite intervention frequency score	0.04	1.13	1.01–1.26
Perceived difficulty in discharging patients to long‐term care facilities (*n* = 283)			
Years of experience as a physician	0.58	1.00	0.97–1.02
Male	0.72	1.11	0.62–2.01
General ward as the primary practice setting	0.33	1.28	0.78–2.09
Experience with home medical care	0.34	0.79	0.50–1.27
Composite intervention frequency score	0.03	1.14	1.02–1.28

Sensitivity analyses yielded results consistent with those of the primary analyses. In the three‐category analysis, the composite intervention frequency score remained significantly associated with perceived difficulty in discharge to home (OR: 1.13, 95% CI: 1.01–1.26, *p* = 0.04) and to long‐term care facilities (OR: 1.14, 95% CI: 1.02–1.28, *p* = 0.02) (Table S4). Similarly, in the multiple imputation analyses, the composite intervention frequency score was associated with perceived difficulty in discharge to home (OR: 1.14, 95% CI: 1.02–1.27, *p* = 0.03) and to long‐term care facilities (OR: 1.13, 95% CI: 1.01–1.27, *p* = 0.03) (Table S5).

## 4 Discussion

In this national survey of hospital‐based geriatricians, most physicians reported difficulty in discharging patients with senility for end‐of‐life care to both home and long‐term care facilities, with a greater degree of difficulty perceived for discharge to home. Regarding the reasons for difficulty, caregiver‐related factors such as insufficient caregiving capacity and strong family preference for hospital care were frequently reported for discharge to home. In contrast, for discharge to long‐term care facilities, facility‐related factors, including limited availability of end‐of‐life care and inability to provide required medical procedures, were more commonly identified. In ordinal logistic regression analyses, a higher tendency to initiate medical interventions in situations such as loss of oral intake was independently associated with greater perceived difficulty in discharge planning. The findings were robust in sensitivity analyses using a three‐category outcome and multiple imputation, which yielded results consistent with those of the primary analyses.

The reasons for perceived difficulty differed according to the discharge setting. For discharge to home, caregiver‐related factors such as insufficient caregiving capacity and strong family preference for hospital care were prominent. These findings are consistent with previous literature. A systematic review has reported that patients and their family caregivers have a range of unmet needs at the end of life, including those related to communication, psychosocial issues, and social isolation [9]. In addition, the other review has shown that family caregivers often feel unprepared and unsupported, which may lead to hospital admission and prevent patients from achieving their preferred place of death [10]. Taken together, these findings suggest that supporting family caregivers and improving their understanding of home‐based end‐of‐life care may be critical to facilitating discharge.

In contrast, for discharge to long‐term care facilities, facility‐related factors such as the inability to provide end‐of‐life care and limitations in performing required medical procedures were more frequently identified. These findings are consistent with previous research indicating that palliative care in Japanese long‐term care facilities remains underdeveloped, with deficits in strategic frameworks, lack of standardized staff training, and limited implementation of quality improvement systems [11]. In addition, the ability of such facilities to provide medical interventions is often constrained by limited staffing and the absence of on‐site physicians, which may restrict feasible discharge destinations. In this context, it may be important for hospital physicians to carefully reconsider the necessity of ongoing medical interventions when planning discharge, particularly when such interventions may limit available care settings.

In the present study, a higher tendency to initiate medical interventions was independently associated with greater perceived difficulty in discharging patients with senility for end‐of‐life care. One possible explanation is that physicians who are more inclined to initiate medical interventions may be influenced by a hospital‐based environment in which relatively intensive care at the end of life may be considered the default approach [12]. While such approaches may be appropriate in acute care settings, they may be less compatible with transitions to community‐based care, where resources and the capacity to provide certain interventions are limited. In this context, it may be important to reconsider the necessity of ongoing medical interventions when planning discharge to ensure alignment with feasible care settings.

This study has several limitations. First, the response rate was moderate, and non‐response bias cannot be excluded. Second, the data were based on self‐reported responses, which may be subject to recall bias and social desirability bias. Because information on non‐respondents was not available, we could not compare the characteristics of respondents with those of the overall target population. Therefore, the direction and magnitude of potential non‐response bias remain uncertain. Third, the study population consisted of board‐certified geriatricians in Japan, which may limit the generalizability of the findings to other physician groups or healthcare systems. Another limitation is that the primary outcome was assessed using a single, self‐reported item that was developed for this study and has not undergone formal psychometric validation. Although the response categories were clearly defined, the measure may not fully capture the multidimensional nature of physicians' perceived difficulty in discharge planning. In addition, the questions assessed only discharge to the same type of residence as before hospitalization (home‐to‐home or facility‐to‐facility) and did not capture transitions to a different setting, such as discharge from home to a long‐term care facility. Therefore, the findings do not capture difficulties associated with changing‐destination transitions and may not fully represent the overall difficulty of discharge planning for patients with senility. Finally, several potentially important hospital‐, patient‐, and regional‐level factors were not collected in the survey and could not be included in the multivariable models. These included hospital size, urban or rural location, the severity and complexity of patients typically treated by the responding physicians, specific medical care needs, non‐clinical barriers such as financial issues, and the availability of home care resources. Therefore, residual confounding by these unmeasured factors cannot be excluded. In addition, because of the cross‐sectional design, the direction of the association between intervention tendency and perceived difficulty cannot be established, and reverse causality cannot be excluded.

## 5 Conclusion

In conclusion, many hospital‐based geriatricians perceived difficulty in discharging patients with senility for end‐of‐life care, particularly for discharge to home. The underlying reasons differed by discharge setting, with caregiver‐related factors being more prominent for home discharge and facility‐related factors for discharge to long‐term care facilities. In addition, a higher tendency to initiate medical interventions was associated with greater perceived difficulty in discharge planning. These findings suggest that both external factors and physicians' clinical approaches may influence end‐of‐life care transitions, highlighting the need for better alignment between hospital care and community‐based care settings.

## Author Contributions

**Teruhiko Imanaga:** conceptualization, investigation, funding acquisition, writing – original draft, writing – review and editing, visualization, validation, methodology, software, formal analysis, project administration, resources, supervision, data curation.

## Funding

This work was supported by the Yuumi Memorial Foundation for Home Health Care (Y2024A104‐005).

## Ethics Statement

This study was approved by the Ethical Review Committee of the Japanese Association for Home Care Medicine (No: 2024‐08).

## Consent

Informed consent was obtained from all participants.

## Conflicts of Interest

The author declares no conflicts of interest.

## Supporting information


**Table S1:** Response options for reasons underlying perceived difficulty in discharging patients with senility.
**Table S2:** Reasons for perceived difficulty in discharge according to experience with home medical care.
**Table S3:** Results of univariable ordinal logistic regression analyses.
**Table S4:** Sensitivity analysis: Multivariable ordinal logistic regression analyses using three‐category outcome for perceived discharge difficulty.
**Table S5:** Sensitivity analysis: Multivariable ordinal logistic regression analyses using multiple imputation for perceived discharge difficulty.

## Data Availability

The data that support the findings of this study are available on request from the corresponding author. The data are not publicly available due to privacy or ethical restrictions.
