# Supplementary material for: Perceived Difficulty in Discharging Older Patients With Senility to Home or Care Facilities for End‐of‐Life Care: A Nationwide Cross‐Sectional Study of Hospital‐Based Geriatricians in Japan
Source: J Gen Fam Med. 2026 Sep 18;27(5):e70181. doi: 10.1002/jgf2.70181 (PMC13589096; doi:10.1002/jgf2.70181)
Supplement: Supplementary file 1 [file JGF2-27-e70181-s001.docx]

TableS1 Response options for reasons underlying perceived difficulty in discharging patients with senility

| Domain | Response option |
| --- | --- |
| Discharge to home | Insufficient caregiving capacity |
|  | Poor family relationships |
|  | Strong patient preference for hospital care |
|  | Strong family preference for hospital care |
|  | Poor patient/family understanding of the condition |
|  | Limited home care resources in the community |
|  | No physicians able to provide required procedures |
|  | Limited in-hospital interdisciplinary collaboration |
|  | Limited community healthcare collaboration |
| Discharge to long-term care facilities | Poor family relationships |
|  | Strong patient preference for hospital care |
|  | Strong family preference for hospital care |
|  | Poor patient/family understanding of the condition |
|  | Facilities not providing end-of-life care |
|  | Facilities unable to provide required procedures |
|  | Limited in-hospital interdisciplinary collaboration |
|  | Limited collaboration with long-term care |

TableS2 Reasons for perceived difficulty in discharge according to experience with home medical care

| discharging to home(n=290) | | | |
| --- | --- | --- | --- |
| Reason | No experience  n (%) | Experience  n (%) | P value |
| Insufficient caregiving capacity | 107 (89.2%) | 144 (85.2%) | 0.38 |
| Poor family relationships | 30 (25.0%) | 42 (24.9%) | 1.00 |
| Strong patient preference for hospital care | 20 (16.7%) | 28 (16.6%) | 1.00 |
| Strong family preference for hospital care | 81 (67.5%) | 111 (65.7%) | 0.80 |
| Poor patient/family understanding of the condition | 51 (42.5%) | 66 (39.1%) | 0.63 |
| Limited home care resources in the community | 49 (40.8%) | 52 (30.8%) | 0.08 |
| No physicians able to provide required procedures | 41 (34.2%) | 39 (23.1%) | 0.045 |
| Limited in-hospital interdisciplinary collaboration | 5 (4.2%) | 5 (3.0%) | 0.75 |
| Limited community healthcare collaboration | 21 (17.5%) | 22 (13.0%) | 0.32 |
| discharging to long-term care facilities (n=275) | | | |
| Reason | No experience  n (%) | Experience  n (%) | P value |
| Poor family relationships | 8 (7.2%) | 6 (3.7%) | 0.26 |
| Strong patient preference for hospital care | 20 (18.0%) | 21 (13%) | 0.30 |
| Strong family preference for hospital care | 55 (49.5%) | 71 (43.8%) | 0.39 |
| Poor patient/family understanding of the condition | 27 (24.3%) | 38 (23.5%) | 0.89 |
| Facilities not providing end-of-life care | 79 (71.2%) | 103 (63.6%) | 0.24 |
| Facilities unable to provide required procedures | 67 (60.4%) | 96 (59.3%) | 0.90 |
| Limited in-hospital interdisciplinary collaboration | 4 (3.6%) | 3 (1.9%) | 0.45 |
| Limited collaboration with long-term care | 25 (22.5%) | 25 (15.4%) | 0.15 |

TableS3 Results of univariable ordinal logistic regression analyses

| Perceived difficulty in discharging patients to home(n=290) | | | |
| --- | --- | --- | --- |
| Variable | P value | Odds ratio | 95% confidence interval |
| Years of experience as a physician | 0.40 | 0.99 | 0.97-1.01 |
| Male | 0.85 | 1.05 | 0.61-1.85 |
| General ward as the primary practice setting | 0.29 | 1.28 | 0.81-2.04 |
| Experience with home medical care | 0.30 | 0.79 | 0.50-1.24 |
| composite intervention frequency score | 0.04 | 1.13 | 1.01-1.26 |
| Perceived difficulty in discharging patients to long-term care facilities (n=283) | | | |
| Variable | P value | Odds ratio | 95% confidence interval |
| Years of experience as a physician | 0.71 | 1.00 | 0.98-1.02 |
| Male | 0.65 | 0.88 | 0.50-1.54 |
| Experience with home medical care | 0.58 | 0.88 | 0.55-1.39 |
| General ward as the primary practice setting | 0.24 | 1.33 | 0.83-2.13 |
| composite intervention frequency score | 0.03 | 1.14 | 1.02-1.28 |

TableS4 Sensitivity analysis: Multivariable ordinal logistic regression analyses using three-category outcome for perceived discharge difficulty

| Perceived difficulty in discharging patients to home(n=290) | | | |
| --- | --- | --- | --- |
| Variable | P value | Odds ratio | 95% confidence interval |
| Years of experience as a physician | 0.43 | 0.99 | 0.97-1.01 |
| Male | 0.60 | 1.17 | 0.65-2.10 |
| General ward as the primary practice setting | 0.48 | 1.19 | 0.73-1.93 |
| Experience with home medical care | 0.28 | 0.89 | 0.49-1.23 |
| composite intervention frequency score | 0.04 | 1.13 | 1.01-1.26 |
| Perceived difficulty in discharging patients to long-term care facilities (n=283) | | | |
| Variable | P value | Odds ratio | 95% confidence interval |
| Years of experience as a physician | 0.57 | 0.99 | 0.97-1.02 |
| Male | 0.72 | 1.11 | 0.62-2.01 |
| General ward as the primary practice setting | 0.36 | 1.26 | 0.77-2.06 |
| Experience with home medical care | 0.32 | 0.79 | 0.49-1.26 |
| composite intervention frequency score | 0.02 | 1.14 | 1.02-1.28 |

TableS5 Sensitivity analysis: Multivariable ordinal logistic regression analyses using multiple imputation for perceived discharge difficulty

| Perceived difficulty in discharging patients to home(n=301) | | | |
| --- | --- | --- | --- |
| Variable | P value | Odds ratio | 95% confidence interval |
| Years of experience as a physician | 0.40 | 0.99 | 0.97-1.01 |
| Male | 0.51 | 1.22 | 0.68-2.18 |
| General ward as the primary practice setting | 0.60 | 1.14 | 0.71-1.84 |
| Experience with home medical care | 0.22 | 0.75 | 0.47-1.19 |
| composite intervention frequency score | 0.03 | 1.14 | 1.02-1.27 |
| Perceived difficulty in discharging patients to long-term care facilities (n=301) | | | |
| Variable | P value | Odds ratio | 95% confidence interval |
| Years of experience as a physician | 0.63 | 0.99 | 0.97-1.02 |
| Male | 0.99 | 1.00 | 0.55-1.79 |
| General ward as the primary practice setting | 0.64 | 0.89 | 0.55-1.44 |
| Experience with home medical care | 0.58 | 0.88 | 0.55-1.39 |
| composite intervention frequency score | 0.03 | 1.13 | 1.01-1.27 |
